# Characteristics, antimicrobial capacity, and antioxidant potential of electrospun zein/polyvinyl alcohol nanofibers containing thymoquinone and electrosprayed resveratrol nanoparticles

**DOI:** 10.1002/fsn3.3816

**Published:** 2023-11-27

**Authors:** Majid Aminzare, Saeideh Soltan Ahmadi, Hassan Hassanzad Azar, Nasser Nikfarjam, Shahin Roohinejad, Ralf Greiner, Reza Tahergorabi

**Affiliations:** ^1^ Department of Food Safety and Hygiene, School of Public Health Zanjan University of Medical Sciences Zanjan Iran; ^2^ Department of Chemistry Institute for Advanced Studies in Basic Sciences Zanjan Iran; ^3^ Division of Food and Nutrition, Burn and Wound Healing Research Center Shiraz University of Medical Sciences Shiraz Iran; ^4^ Department of Food Technology and Bioprocess Engineering, Max Rubner‐Institut Federal Research Institute of Nutrition and Food Karlsruhe Germany; ^5^ Food and Nutritional Sciences Program North Carolina Agricultural and Technical State University Greensboro North Carolina USA

**Keywords:** antibacterial, antioxidant, bioactive compound, nanofiber, nanoparticle

## Abstract

The aim of the present study was to fabricate, characterize, and evaluate the in vitro antimicrobial and antioxidant properties of zein/polyvinyl alcohol (ZN/PVA) nanofibers containing 2% and 4% of thymoquinone (TQ), either alone or in combination with electrosprayed ZN nanoparticles containing 1% and 2% of resveratrol (RS). According to scanning electron microscopy analysis, the diameter of nanofibers and nanoparticles increased with increasing TQ and RS concentrations, respectively. The molecular interaction between ZN or PVA polymers and TQ or RS was confirmed by Fourier transform infrared spectroscopy. Thermogravimetric analysis showed that the thermal stability of nanofibers did not change with the addition of TQ and RS. Moreover, incorporation of TQ in nanofibers along with RS nanoparticles increased their antibacterial and free radical scavenging activities based on broth dilution and DPPH methods, respectively (*p* ≤ .05). *Escherichia coli* O157:H7 (as a Gram‐negative pathogenic bacteria) was more resistant to all treatments than *Staphylococcus aureus* (as a Gram‐positive pathogenic bacteria). In addition, the combined use of TQ in nanofibers and RS nanoparticles had antagonistic antibacterial and synergistic antioxidant effects. The best results were obtained with ZN/PVA nanofiber containing 4% TQ and electrosprayed with 2% RS nanoparticles (*p* ≤ .05). According to the results of the present study, biodegradable ZN/PVA nanofiber containing TQ and electrosprayed with RS nanoparticles can be used as a novel active packaging material in the food industry.

## Graphical abstract

ZN/PVA nanofibers containing 2% and 4% of TQ were prepared by the electrospinning method. ZN/PVA nanofibers were electrosprayed with ZN nanoparticles containing 1% and 2% of RS. Nanofibers were characterized by SEM, IR, and TGA methods. The in vitro antioxidant and antibacterial effects of nanofibers were compared. ZN/PVA TQ4RS2 nanofiber had the highest in vitro antioxidant and antibacterial activity.

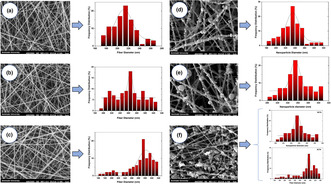

## 1 INTRODUCTION

Foodborne infections and intoxications caused by pathogenic bacteria have always been considered as major public health concerns. Despite the efforts of regulatory bodies, scientists, and industrialists, foodborne pathogens continue to cause significant damage to national economies and the social welfare of populations. Pathogenic strains of *Escherichia coli* and *Staphylococcus aureus* can compromise the microbial safety of food products due to improper preparation and distribution practices (Haghi et al., 2019; Lee & Yoon, 2021; Newell et al., 2010; Soltaninezhad et al., 2020). Another concern that threatens consumer safety is the oxidation process in foods. Lipid oxidation reduces the sensory and nutritional properties of the product, causes economic loss, and causes cardiovascular diseases and cancer (Grootveld et al., 2020; Nieva‐Echevarría et al., 2020). In this regard, the use of antioxidant and antimicrobial compounds is a suitable solution for improving the quality of food products and ensuring consumer safety. Nevertheless, due to evidence of the adverse effects of synthetic preservatives on human health, several valuable research studies have been conducted to replace them with natural alternatives, such as plant‐based preservatives (Davidson et al., 2015; Hosseini et al., 2019; Shahidi & Ambigaipalan, 2015). However, one of the main limitations of the use of plant‐based preservatives is the negative sensory effects they can have on food products at the high concentrations required. The combined use of these preservatives can enhance their effectiveness at lower concentrations through possible synergistic interactions, thereby reducing their negative sensory effects (Bag & Chattopadhyay, 2015). Thymoquinone (TQ), the main compound found in black cumin (*Nigella sativa*), is a monoterpenoid with antioxidant and antimicrobial properties (Talebi et al., 2021; Yousefizadeh, Aminzare, & Hassanzadazar, 2022). Resveratrol (RS) is a polyphenolic stilbene compound found in peanuts, strawberries, and red grapes that possesses several health benefits, including antioxidant and antimicrobial properties (Gerszon et al., 2014; Ma et al., 2018).

The direct addition of natural preservatives to food products can lead to their rapid consumption and subsequent loss of their protective effects. One of the most successful approaches to solving this problem is the use of active packaging systems. Through this approach, antimicrobial and antioxidant compounds are released from the packaging material into the food at a controlled rate (Charles et al., 2021). Today, the food packaging industry primarily relies on synthetic polymers due to their favorable properties, including ease of production, flexibility, and stability (Yadav et al., 2018). However, synthetic polymers pose several health problems for consumers, such as the transfer of solvent residues and monomers to food products, as well as environmental issues, including non‐recyclability, the emission of toxic gases, and their contribution to global warming. As a result, the growing demand from the global community has led to the replacement of synthetic polymers with biodegradable alternatives (Mignon et al., 2019). In this context, different biopolymers and biocompatible polymers have been suggested for electrospun active food packaging systems. Zein (ZN), a biopolymer derived from maize seeds' storage protein, has garnered significant interest due to its affordability and lack of toxicity (Moradkhannejhad et al., 2018). However, due to the dry and brittle nature of electrospun zein nanofibers, blending them with other polymers can enhance their mechanical performance. Polyvinyl alcohol (PVA), a biocompatible polymer, can be added to the zein electrospinning solution to enhance the dry mechanical properties of the nanofibers (Zhang et al., 2014).

Electrohydrodynamic processing, which includes techniques such as electrospinning and electrospraying, is a method of carrying bioactive materials that is commonly used in the construction of active food packaging systems. Electrospinning is utilized to produce nanofibers, while electrospraying is used to generate nanoparticles (Charles et al., 2021; Zhang et al., 2020). Nanofibers offer numerous advantages over other active food packaging films. Their high porosity allows for greater loading capacities for bioactive compounds. Additionally, electrospun nanofibers possess a high surface‐to‐volume ratio, enhancing the effectiveness and sustained release of these bioactive compounds. Another benefit is that electrospinning is a non‐thermal process, ensuring that heat‐sensitive bioactive compounds incorporated into the packaging materials remain unaffected and retain their functional properties (Charles et al., 2021; Tang et al., 2019; Zhang et al., 2020). Electrospraying is a one‐step process that enables the production of dry nanoencapsulates and demonstrates excellent performance under ambient temperature and atmospheric pressure. Due to these advantages, it is considered a suitable method for incorporating bioactive compounds into active food packaging materials. By encapsulating resveratrol within zein protein using electrospray technology, it is possible to protect the compound from environmental conditions and enhance its water solubility (Jayan et al., 2019).

Based on our literature review, several studies have assessed the in vitro antibacterial and antioxidant effects of electrospun nanofibers or electrosprayed nanoparticles containing TQ or RS (Gomaa et al., 2017; Jayan et al., 2019; Leena et al., 2020; Li et al., 2020). However, to the best of our knowledge, no research has utilized a combination of electrospraying and electrospinning techniques to produce active food packaging materials incorporating the aforementioned natural preservatives. Therefore, the objective of the present study was to (1) fabricate and characterize ZN/PVA nanofibers containing varying concentrations of TQ, with or without ZN nanoparticles containing different concentrations of RS; (2) evaluate and compare their antibacterial and antioxidant potential; and (3) assess the interactions between TQ and RS using an in vitro model.

## 2 MATERIALS AND METHODS

### 2.1 Materials

Thymoquinone (2‐isopropyl‐5‐methyl‐1,4‐benzoquinone), resveratrol (3,4′,5‐trihydroxy‐*trans*‐stilbene), glacial acetic acid, 1,1‐diphenyl‐2‐picrylhydrazyl (DPPH), ethanol, and methanol were purchased from Sigma Aldrich Company. Purified zein powder (90.3% protein content) was purchased from Acros Organics and used without further purification. Merck Company supplied PVA (Mw: 72,000 g/mol), nutrient agar, brain heart infusion (BHI) broth, and BHI agar for this study. The freeze‐dried vials of *S. aureus* (PTCC 1431) and *E. coli* O157:H7 (PTCC 1860) were purchased from the microbial collection at the Iranian Research Organization for Science and Technology, located in Tehran, Iran.

### 2.2 Preparation of feed solutions

To prepare electrospinning solutions, 22.5 g of ZN powder was dissolved in 70 mL of glacial acetic acid. Then, 7.5 g of PVA powder were added to the solution to obtain a 30% (w/v) polymer solution. After cooling, different amounts of TQ (0.6 and 1.2 g) were slowly added to the ZN/PVA solutions with continuous stirring. The solutions were kept at 25°C for 24 h to remove any probable air bubbles and used to prepare ZN/PVA nanofibers containing 2 and 4% TQ (w/w, with respect to ZN/PVA). To prepare the control group, TQ was not added to the ZN/PVA solution (Teilaghi et al., 2020; Zhang et al., 2014).

To prepare electrospraying solutions, 5% ZN powder was dissolved in 80% ethanol with continuous stirring. Then, 50 and 100 mg of RS were added separately to the mentioned solutions. These solutions were used to prepare ZN nanoparticles containing 1% and 2% RS (w/w, with respect to ZN; Bhushani et al., 2017; Jayan et al., 2019).

### 2.3 Electrospinning and electrospraying

An electrospinning device (ES1000 model, Fanavaran Nano‐Meghyas Co.) with a flow rate of 0.1 mL/h, an applied voltage of 25 kV, and a 150‐mm distance between the syringe and collector was used for the preparation of nanofibers (Khalifezadeh & Shahabi, 2019; Teilaghi et al., 2020; Zhang et al., 2014). The electrospinning solutions were filled into 5 mL plastic syringes connected with a stainless‐steel nozzle. A spinning drum covered with aluminum foil caught the fibers at 1000 rpm. Electrospinning was performed at ambient temperatures and humidity. To eliminate solvent residues, the nanofiber samples were dried in a vacuum oven for 24 h at 25°C.

For nanoparticle preparation, electrospraying solutions were separately filled into a 5‐mL plastic syringe and electrosprayed by a stainless‐steel nozzle connected to a 30 kV voltage power source onto the nanofiber substrate prepared above. The feed flow rate and the distance between the needle tip and collector were 0.5 mL/h and 80 mm, respectively (Jayan et al., 2019). The list of all treatments is shown in Table 1.

**TABLE 1:** The list of treatments.

No.	Treatment	Description
1	ZN/PVA	Zein/polyvinyl alcohol nanofiber
2	ZN/PVA‐TQ2	Zein/polyvinyl alcohol nanofiber containing 2% thymoquinone
3	ZN/PVA‐TQ4	Zein/polyvinyl alcohol nanofiber containing 4% thymoquinone
4	ZN/PVA‐RS1	Zein/polyvinyl alcohol nanofibers electrosprayed with zein nanoparticles containing 1% resveratrol
5	ZN/PVA‐RS2	Zein/polyvinyl alcohol nanofibers electrosprayed with zein nanoparticles containing 2% resveratrol
6	ZN/PVA‐TQ2‐RS1	Zein/polyvinyl alcohol nanofiber containing 2% thymoquinone and electrosprayed with zein nanoparticles containing 1% resveratrol
7	ZN/PVA‐TQ2‐RS2	Zein/polyvinyl alcohol nanofiber containing 2% thymoquinone and electrosprayed with zein nanoparticles containing 2% resveratrol
8	ZN/PVA‐TQ4‐RS1	Zein/polyvinyl alcohol nanofiber containing 4% thymoquinone and electrosprayed with zein nanoparticles containing 1% resveratrol
9	ZN/PVA‐TQ4‐RS2	Zein/polyvinyl alcohol nanofiber containing 4% thymoquinone and electrosprayed with zein nanoparticles containing 2% resveratrol

### 2.4 Characterization of nanofibers

#### 2.4.1 Scanning electron microscopy (SEM)

In order to investigate the morphology of nanofibers and nanoparticles, a scanning electron microscope (SEM: Quanta 200; FEI) was used. The SEM device operated at 25 kV and images were recorded at 1000 to 25,000× magnification range. Samples were coated with gold prior to imaging. The size and diameter distribution of nanofibers and nanoparticles were measured using JMicrovision software (version 1.2.7 for Windows) in at least 100 randomly selected locations in SEM images. The following equation was used to compute the average size of nanofibers and nanoparticles:

$$\;\bar{d}=\sum n_{i}d_{i}/\sum n_{i}$$

where *n*_*i*_ is the number of fibers having *d*_*i*_ diameters (Moradkhannejhad et al., 2018).

#### 2.4.2 Fourier transform infrared spectroscopy (FT‐IR)

The Fourier transform infrared spectroscopy (FT‐IR) spectrometer (Nicolet IS10; Thermo Scientific) was used to identify the interaction and the bonds formed between ZN, PVA, TQ, and RS. For this purpose, the control treatment, the single treatments containing the highest concentration of each of the bioactive compounds, and their combined treatment were selected. The KBr plates were prepared before analysis, and the spectra were recorded from 400 to 4000 cm^−1^ with 4 cm^−1^ resolution, averaging a minimum of 16 scans (Moradkhannejhad et al., 2018).

#### 2.4.3 Thermogravimetric analysis (TGA)

A thermogravimetric analyzer (Q600 SDT; TA Instruments) was used to test the thermal characteristics of ZN/PVA nanofibers. The samples (5 mg) were heated in platinum capsules in the temperature range of 25–600°C at a heating rate of 10°C/min under a nitrogen flow of 50 mL/min. As a reference, an empty platinum plate was employed. The weight loss of nanofibers (%) was specified from the TGA curve, and the maximum decomposition temperature was obtained from a derivative form of the TGA (DTG) curve (Shankar et al., 2019).

### 2.5 Evaluation of antibacterial properties

The antibacterial activities of nanofibers against *S. aureus* (Gram‐positive bacteria) and *E. coli* O157:H7 (Gram‐negative bacteria) were evaluated using the broth dilution method. In order to prepare the studied bacterial strains, initially, the stock bacteria were inoculated in 15 mL of BHI broth and incubated at 37°C for 24 h at 150 rpm using a shaker incubator (model A.R 82; Pars Azma Co.). Then, inoculums were separated from broth via centrifuging at 4024 *g* for 5 min. During the last two steps of centrifugation, physiological saline replaced the supernatant. Different dilutions of bacterial suspensions were then prepared, and their absorbance was read at 600 nm using a spectrophotometer (DR 5000; HACH Co.) to adjust a suitable dilution of bacteria (~1–2 × 10^5^ CFU/mL). In order to confirm the results, bacterial counts were performed on BHI agar at 37°C for 24 h (Abdollahzadeh et al., 2014).

To evaluate the antibacterial properties of the samples, 25 mg of each nanofiber was transferred to the test tubes containing 10 mL of bacterial suspension and incubated at 37°C for 24 h using a shaker incubator. Then, 0.1 mL of each tube was cultured on nutrient agar plates and incubated at 37°C for 24 h. Test tubes without bacteria and nanofiber were used as negative and positive controls, respectively. Bacterial counts were reported as log_10_ CFU/mL (Sugumar et al., 2015).

To calculate the bacterial reduction index, the positive controls were enumerated immediately after preparing the suspension (CON‐0) and again after 24 h of incubation (CON‐24), following the guidelines outlined by the International Organization for Standardization (ISO) method. The formula provided was used in this calculation (ISO‐22196, 2007):

$$\text{Bacterial reduction index}=\left[\log\;\left(B/A\right)–\log\;\left(C/A\right)\right]=\log\;\left(B/C\right)$$

where *A* is the bacterial count of the control sample immediately after the suspension preparation (CON‐0), *B* is the bacterial count of the control sample after 24 h incubation (CON‐24), and *C* is the bacterial count of the nanofiber samples after 24 h incubation.

The antibacterial activities of nanofibers were determined as follows (Torres‐Giner et al., 2017):

Nonsignificant: 0.5 > Bacterial reduction index;
Slight: 1 > Bacterial reduction index ≥0.5;
Significant: 3 > Bacterial reduction index ≥1;
Strong: 3 ≤ Bacterial reduction index.

The percentage of inhibition in bacterial growth was determined using the specified formula:

$$\text{Percentage of inhibition}\;\left(\%\right)=\left(\mathrm{CON}–\text{Treatment}/\mathrm{CON}\right)\times 100$$

where CON is the mean of bacterial counts of the control sample after 24 h incubation (CON‐24), and treatment is the mean of bacterial counts of the treated samples after 24 h incubation.

To determine the antibacterial interactions between ZN/PVA‐TQ and ZN/PVA‐RS samples, the inhibition percentage of ZN/PVA‐TQ samples was statistically added to the inhibition percentage of ZN/PVA‐RS samples and recorded as “expected inhibition percentage”. Then, the evaluated inhibition percentage of samples containing a combination of TQ and RS (ZN/PVA‐TQ‐RS) at similar concentrations was compared with them. The expected inhibition percentage greater than, smaller than, or equal to the inhibition percentage of samples containing a combination of TQ and RS indicates synergistic, antagonistic, or additive effects, respectively. For this purpose, decimals in log_10_ CFU/mL values were not considered (Aminzare et al., 2022).

### 2.6 Evaluation of antioxidant properties

The antioxidant activity of nanofibers was evaluated by the DPPH free radical scavenging activity method. 25 mg of each sample was dissolved in 3 mL of distilled water. Then, 2.8 mL of the mentioned solution was added to the test tubes containing 0.2 mL of DPPH methanolic solution (10^−3^ M) and incubated at 25°C for 60 min. Finally, the absorbance of the solutions was measured at 517 nm using a spectrophotometer. The DPPH absorbance was considered 100%, and the percentage of antioxidant activity was estimated using the following equation:

$$\text{Antioxidant activity}\;\left(\%\right)=\frac{\left(A_{C}-A_{s}\right)}{\mathrm{Ac}}\times 100$$

where *A*_c_ is the absorbance of control DPPH and *A*_s_ is the absorbance of nanofiber samples.

The combination index was used to calculate the antioxidant interactions between RS and TQ in nanofiber samples with the following formula:

$$\text{Combination index}=\frac{\frac{\mathrm{DPP}H_{\mathrm{ab}}}{2}}{\mathrm{DPP}H_{a}}+\frac{\frac{\mathrm{DPP}H_{\mathrm{ab}}}{2}}{\mathrm{DPP}H_{b}}$$

where DPPH_ab_ values are the radical scavenging activities of ZN/PVA‐TQ‐RS nanofibers. DPPH_a_ and DPPH_b_ values are the radical scavenging activities of ZN/PVA‐TQ and ZN/PVA‐RS, respectively. Synergistic, additive, or antagonistic effects are indicated by greater than, equal to, or smaller than 1 combination indices, respectively (Hashemi et al., 2019).

### 2.7 Statistical analysis

SPSS software (V.18 for Windows; SPSS Inc.) and one‐way analysis of variance (ANOVA) along with Tukey's honest significant difference post hoc test were used to analyze the data (*α* = 0.05). All tests were performed in technical triplicate, and results were reported as “Mean ± SE.”

## 3 RESULTS AND DISCUSSION

### 3.1 Morphology analysis

**FIGURE 1:**
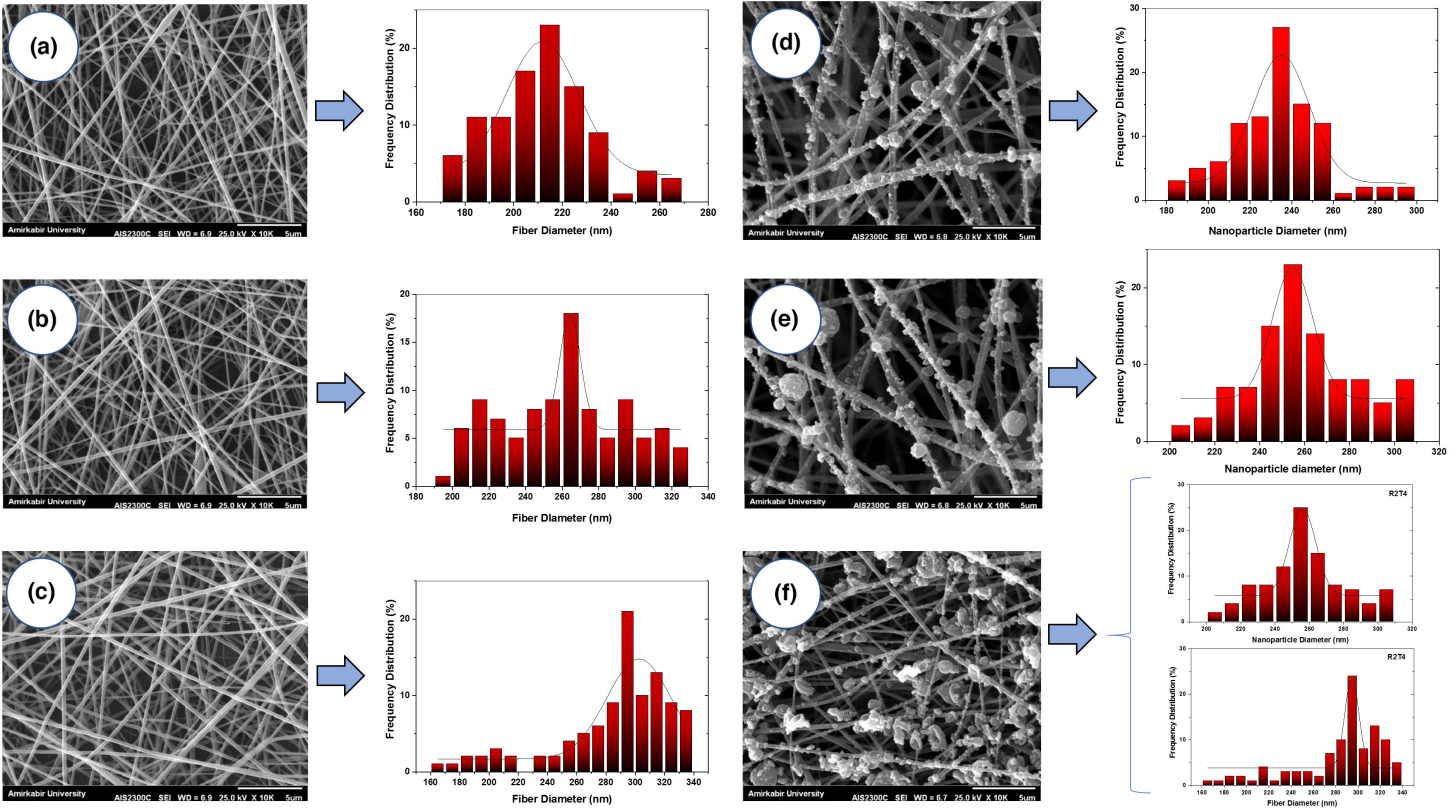
SEM micrographs and related diameter distribution of nanofibers and nanoparticles. The scale marker in the figures is 5 μm. (a) ZN/PVA, (b) ZN/PVA‐TQ2, (c) ZN/PVA‐TQ4, (d) ZN/PVA‐RS1, (e) ZN/PVA‐RS2, and (f) ZN/PVA‐TQ4‐RS2.

The morphology and diameter of electrosprayed nanoparticles and electrospun nanofibers containing bioactive compounds can influence their properties and applications in active packaging systems, such as the protection of various bioactive agents as well as mechanical and barrier properties (Jayan et al., 2019; Moradkhannejhad et al., 2018; Zhang et al., 2020). Figure 1 indicates the SEM scans and related diameter distributions of electrospun ZN/PVA nanofibers containing different concentrations of TQ and electrosprayed ZN nanoparticles containing different concentrations of RS. According to Figure 1a–c, the nanofibers formed a network of homogeneous, rod‐shaped, and smooth fibers without any beads, crystals, branches, or links. The average diameter of ZN/PVA nanofiber was 212.1 nm, which was lower than previously reported for ZN/PVA nanofibers. This difference may be due to the ratio of ZN to PVA in the electrospinning solution (Zhang et al., 2014). Duration of nanofiber preparation, distance from nozzle to collector, applied electric field, feeding rate, viscosity of the polymer solution, polymer concentration and conformation, solvent type and its evaporation, and ionic conductivity are other factors that can affect the electrospun nanofiber diameter. (Moradkhannejhad et al., 2018; Zhang et al., 2014). After adding 2 and 4% TQ (w/w, with respect to ZN/PVA) to the electrospinning solution, the diameter of nanofibers increased to 261.3 and 286.1 nm, respectively (*p* ≤ .05). The interaction between ZN and/or PVA and the bioactive compound probably reduced the polyelectrolyte properties and ionic conductivity of the polymer solution as well as the repulsion between the polycationic protein chains and subsequently increased the diameter of the nanofibers (Moradkhannejhad et al., 2018; Najafi et al., 2022). Also, adding the bioactive compound can increase the viscosity of the polymer solution and prevent elongation, which also leads to the creation of fibers with a thicker diameter (Shen et al., 2021). Other studies have already confirmed that the diameter of nanofibers based on electrospun ZN or PVA increases with the increase in the bioactive compound concentration (Leena et al., 2020; Moradkhannejhad et al., 2018; Najafi et al., 2022; Shen et al., 2021; Teilaghi et al., 2020).

As shown in Figure 1d,e, the electrosprayed nanoparticles had a smooth and spherical surface. The smooth surface of nanoparticles can be due to the high ability of ZN film formation. Also, when the polymer concentration is sufficient, spherical particles are produced during the electrospraying process due to intermolecular interactions within the polymer chain (Bhushani et al., 2017; Jayan et al., 2019). The average particle sizes of ZN nanoparticles containing 1 and 2% RS (w/w, with respect to ZN) were 233.2 and 259.2 nm, respectively. Thus, the diameter of ZN nanoparticles increased with increasing RS concentration. The increase in the nanoparticle size may be due to the change in the viscosity of the ZN solution (Jayan et al., 2019). A previous study on electrosprayed ZN nanoparticles containing different concentrations of RS confirms the present findings (Jayan et al., 2019). Also, similar changes in nanoparticle diameters of electrosprayed ZN nanoparticles after loading different concentrations of catechins have been reported in another study (Bhushani et al., 2017). Moreover, Figure 1f indicates the morphology and related diameter distribution of nanofibers and nanoparticles in the ZN/PVA‐TQ4‐RS2 sample. The average diameters of nanofibers and nanoparticles in this sample were 284.4 and 257.5 nm, respectively, confirming the obtained diameters of ZN/PVA nanofibers containing 4% TQ (Figure 1c) and ZN nanoparticles containing 2% RS (Figure 1e).

### 3.2 FT‐IR of nanofibers

**FIGURE 2:**
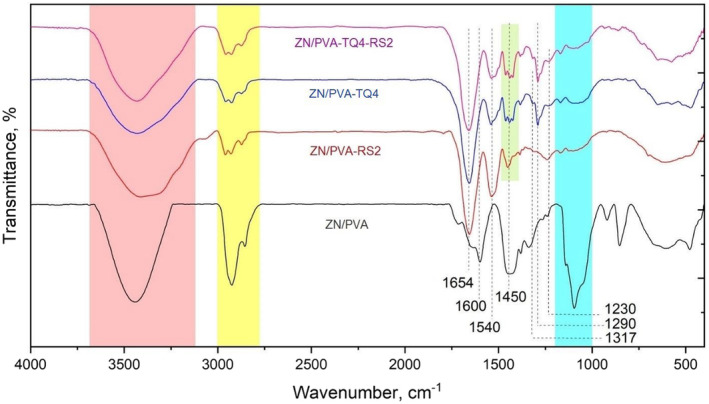
FT‐IR spectra of different nanofibers.

The electrospun nanofibers were subjected to FT‐IR spectroscopy in order to characterize the functional groups as well as to ascertain the nature of the reaction and the bonds formed between ZN/PVA and other ingredients. The FT‐IR spectra of prepared nanofibers are shown in Figure 2. For the ZN/PVA sample, the broad peak at 3250–3650 cm^−1^ is assigned to stretching vibration of O‐H of hydroxyl groups in the PVA and ZN and O‐H of carboxylic groups in the ZN. The peaks at 2850–2950 cm^−1^ are attributed to the stretching vibration of aliphatic C‐H bonds in the ZN and PVA. The peaks at 1000–1200 cm^−1^ are related to the stretching vibration of C‐O bonds in the PVA and ZN structures (Coates, 2000). The peaks at 1654 and 1540 cm^−1^ are related to the stretching vibration of the C=O bond of amid I and bending vibration of N‐H bond of amide II in the ZN, respectively. The peaks at 1317, 1290, and 1230 cm^−1^ can be assigned to the stretching vibration of the C‐N bond of amide III of ZN. Also, the peaks at 1600, 1515, and 1450 cm^−1^ are related to the stretching vibration of aromatic C=C of phenyl groups in the ZN (Najafi et al., 2022; Zhang et al., 2014). All these peaks can be observed in the ZN/PVA nanofibers containing TQ and electrosprayed RS nanoparticles, with some shifts and overlapping. For example, by incorporating TQ and RS in samples, the area under the peak at 3300–3600 cm^−1^ was broadened due to the addition of new hydroxyl groups and their incorporation with PVA and ZN functional groups. Also, the peak of 1450 cm^−1^ (related to the aromatic C=C bond) is split into multiple peaks, showing the presence of newly added aromatic‐based structures to the ZN/PVA matrix. Moreover, the peaks related to the aliphatic C‐H bond are again split into multiple peaks, showing the addition of new structures containing aliphatic groups to the ZN/PVA matrix. Other studies have also shown similar FT‐IR spectra of ZN or PVA nanofiber containing different concentrations of *Nigella sativa* extract (containing TQ as the main compound) (Ali et al., 2021; Teilaghi et al., 2020), electrosprayed ZN nanoparticles containing different concentrations of RS (Jayan et al., 2019), ZN/PVA nanofiber (Zhang et al., 2014), and ZN nanofiber containing different concentrations of RS (Leena et al., 2020).

### 3.3 Thermal properties of nanofibers

**FIGURE 3:**
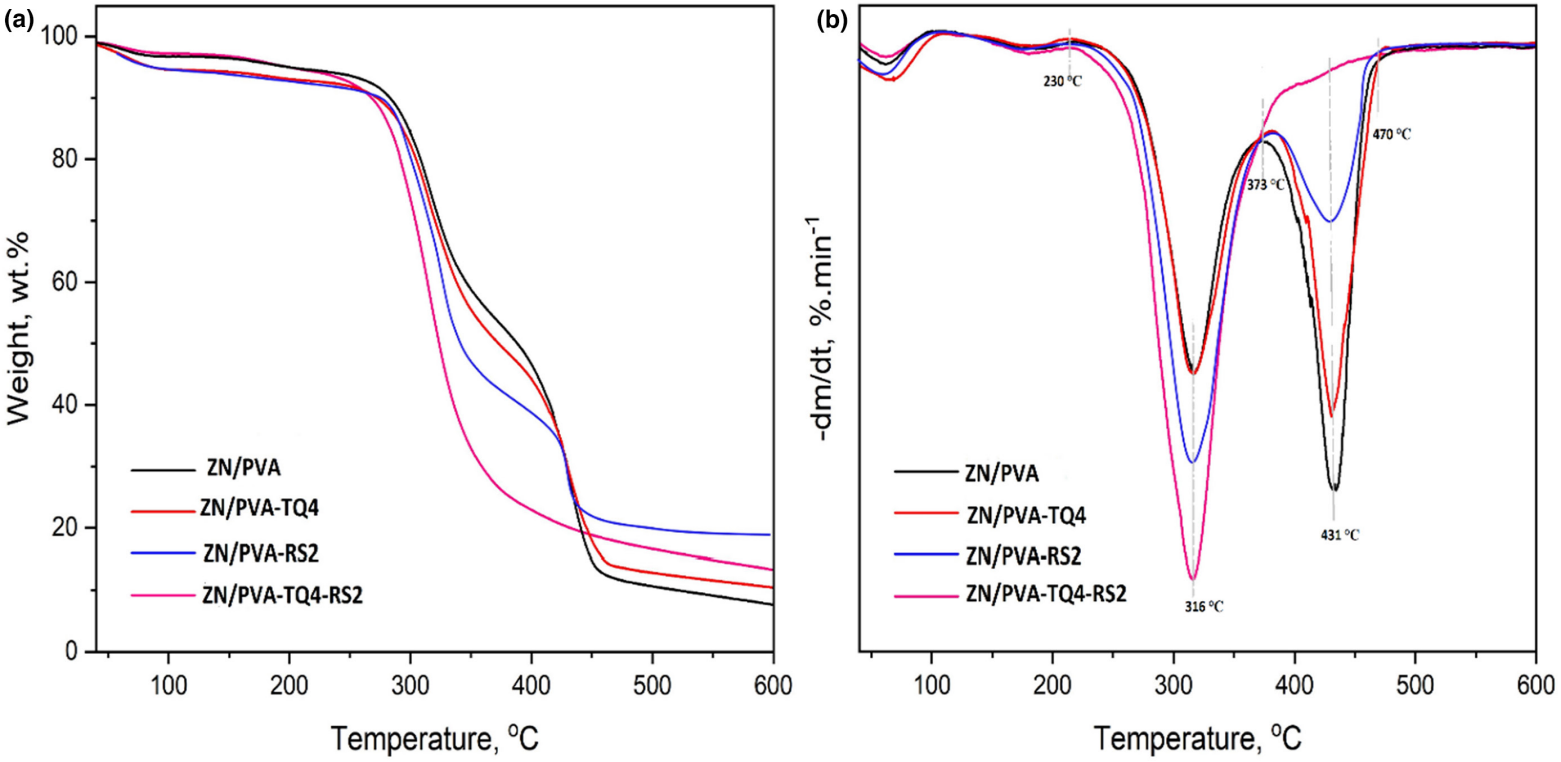
(a) TGA and (b) DTG curves of different nanofibers.

The thermal characteristics of nanofibers were investigated using the TGA technique (Figure 3). This analysis shows the effects of adding TQ and/or RS on the thermal degradation of ZN/PVA nanofibers. Samples were perfectly stable up to 230°C and showed no signs of significant degradation. However, some small peaks around 100°C are probably related to water loss. Sample thermograms revealed two major weight decreases between 200 and 500°C. The first degradation stage was observed at 230–373°C with a maximum degradation at 316°C, which was consistent with the reported temperature degradation range for ZN as well as ester units and side chains of PVA in ZN/PVA nanofibers (Cui et al., 2015; Ding et al., 2010; Kayaci & Uyar, 2012). Also, at this stage, PVA is probably dehydrated, and a polystyrene‐like structure is formed due to the removal of H_2_O from the polymer (Mallakpour & Dinari, 2013). The second degradation stage was observed at 373–470°C with a maximum degradation at 431°C, which can be attributed to the separation of intermolecular and intramolecular forces with partial degradation of the PVA backbone (Cui et al., 2015). In addition, this stage of degradation may be related to the decomposition of the main chain of PVA, including the further degradation of polyethylene‐like residues formed in the first degradation stage and the subsequent formation of carbon and hydrocarbons (Ding et al., 2010; Mallakpour & Dinari, 2013). As can be seen, the first weight loss was greater than the second one for ZN/PVA‐RS2 and ZN/PVA‐TQ4‐RS2 samples, which can be attributed to the increase in ZN weight after electrospraying ZN nanoparticles containing RS on nanofiber mats. Moreover, since RS has two temperature degradation ranges close to the mentioned temperature degradation ranges observed in this study (Marinheiro et al., 2021), it seems that the weight loss of RS overlaps with the weight loss of ZN and PVA. However, hydrogen bonding between RS and the amino groups of proline in ZN (as a rich source of prolamin protein) probably caused the major decomposition of RS in the first degradation stage along with the ZN polymer (Shen et al., 2021). On the other hand, according to previous studies, TQ has a degradation stage at 65–213°C (Al‐Qubaisi et al., 2019; Pagola et al., 2004). But, since no significant weight loss was observed for the samples containing TQ (ZN/PVA‐TQ4 and ZN/PVA‐TQ4‐RS2), it can be concluded that the interaction of TQ with ZN and PVA caused its degradation to overlap with the first main weight loss. The TGA graph (Figure 3a) shows that the addition of ingredients to the pure polymers can improve the thermal features of the nanofibers at temperatures higher than ~450°C; however, no significant changes were observed among the groups. In agreement with these results, Ullah et al. (2019) reported a similar offset of the thermal deterioration curve of ZN nanofibers with and without AgSD at the same temperatures, and there were hardly any noticeable effects of adding AgSD on the thermal stability of pure ZN nanofibers. Furthermore, the existence of evidence of ZN, PVA, TQ, and RS in the final structure can confirm that the ZN/PVA solution did not experience any phase separation and ZN content loss during the electrospinning process. As a polyelectrolyte material, ZN is susceptible to phase separation and fluidic jet branching due to the higher surface charge density applied during the electrospinning process (Chen et al., 2022). All these can lead to loose ZN content in the final electrosprayed ZN/RS nanoparticle‐decorated ZN/PVA/TQ nanofiber.

### 3.4 In vitro antibacterial and antioxidant properties of ZN/PVA nanofibers

#### 3.4.1 Antibacterial properties

The results of the antibacterial properties of ZN/PVA nanofibers loaded with different concentrations of TQ and/or electrosprayed RS nanoparticles against *S. aureus* and *E. coli* O157:H7 are presented in Table 2. The antibacterial potential of the nanofibers followed a concentration‐dependent manner, so that the addition of TQ with or without 2% RS nanoparticles to ZN/PVA nanofibers significantly reduced the population of all studied bacteria (*p* ≤ .05). All single or combined treatments containing 4% TQ were significantly able to reduce the initial bacterial populations (CON‐0) (*p* ≤ .05). Moreover, the inhibitory effects of combined treatments were greater than those of single treatments on bacterial growth. However, antagonistic effects between TQ and RS were observed in all ZN/PVA‐TQ‐RS samples. In other words, the “expected inhibition percentage” was lower than the observed inhibition percentage in the mentioned nanofibers. The highest antibacterial activity was observed for the ZN/PVA‐TQ4‐RS2 treatment, with bacterial counts of 4.99 and 5.19 log CFU/mL (*p* ≤ .05), bacterial reduction indices of 1.78 and 1.52 (significant antibacterial activities), and inhibition percentages of 26.27% and 22.66% against *S. aureus* and *E. coli* O157:H7, respectively. In addition, *E. coli* O157:H7, as a Gram‐negative bacteria, was more resistant than *S. aureus*, as a Gram‐positive bacteria, against all treatments. In a study conducted by Li et al. (2020), the in vitro antibacterial activity of gelatin/zein nanofibers containing different concentrations of RS followed a concentration‐dependent manner against *S. aureus* and *E. coli*. Moreover, Gomaa et al. (2017) reported the similar in vitro antibacterial activities of polylactic acid/cellulose acetate nanofibers containing TQ against the mentioned bacteria. In addition, Ali et al. (2021) demonstrated the similar in vitro antibacterial properties of a PVA nanofibrous mat containing *Nigella sativa* extract against *S. aureus* and *E. coli*. They claimed that the antibacterial activities of PVA nanofibers may be due to the presence of TQ as the main compound in *Nigella sativa* extract. Also, other studies have demonstrated the similar in vitro antibacterial behavior of food packaging materials containing TQ (Yousefizadeh, Hassanzadazar, & Aminzare, 2022) or RS (Aminzare et al., 2022) against *S. aureus* and *E. coli*. All mentioned studies concluded that *S. aureus* (Gram‐positive bacteria) was more susceptible to nanofibers or food packaging materials containing TQ or RS compared with *E. coli* (Gram‐negative bacteria), which confirms the results of the present study.

**TABLE 2:** Antibacterial activity of ZN/PVA nanofibers incorporated with TQ and/or electrosprayed RS nanoparticles (Mean ± SE).

Sample	*Staphylococcus aureus*			*Escherichia coli* O157:H7		
	Bacterial count (log CFU/mL)	Bacterial reduction index	Percentage of inhibition (%)	Bacterial count (log CFU/mL)	Bacterial reduction index	Percentage of inhibition (%)
CON‐0	5.51 ± 0.02^f^	–	–	5.49 ± 0.02^e^	–	–
CON‐24	6.76 ± 0.01^a^	–	–	6.71 ± 0.01^a^	–	–
ZN/PVA	6.75 ± 0.01^a^	0.01 (−)	0.15	6.70 ± 0.02^a^	0.01 (−)	0.13
ZN/PVA‐TQ2	6.02 ± 0.02^d^	0.75 (+)	11.04	6.35 ± 0.01^c^	0.36 (−)	5.39
ZN/PVA‐TQ4	5.22 ± 0.01^g^	1.55 (++)	22.87	5.29 ± 0.01^f^	1.43 (++)	21.25
ZN/PVA‐RS1	6.63 ± 0.04^b^	0.14 (−)	2.06	6.64 ± 0.03^a^	0.07 (−)	1.07
ZN/PVA‐RS2	6.36 ± 0.03^c^	0.40 (−)	6.00	6.53 ± 0.01^b^	0.18 (−)	2.70
ZN/PVA‐TQ2‐RS1	5.95 ± 0.01^d^	0.82 (+)	12.09 (A)	6.31 ± 0.01^c^	0.40 (−)	5.99 (A)
ZN/PVA‐TQ2‐RS2	5.64 ± 0.02^e^	1.12 (++)	16.55 (A)	6.22 ± 0.01^d^	0.49 (−)	7.31 (A)
ZN/PVA‐TQ4‐RS1	5.16 ± 0.02^g^	1.60 (++)	23.65 (A)	5.32 ± 0.01^f^	1.39 (++)	20.71 (A)
ZN/PVA‐TQ4‐RS2	4.99 ± 0.03^h^	1.78 (++)	26.27 (A)	5.19 ± 0.02^g^	1.52 (++)	22.66 (A)

*Note*: Values followed by different letters within the same columns are significantly different according to the Tukey's test (*p* ≤ .05). (−): Nonsignificant antibacterial activity; (+): Slight antibacterial activity; (++): Significant antibacterial activity; (+++): Strong antibacterial activity.

Abbreviations: A, Antagonistic effect; Ad, Additive effect; S, Synergistic effect.

TQ's antibacterial activity is mediated by the formation of reactive oxygen species (ROS), which cause permanent damage to bacterial DNA, proteins, and membranes, causing cell death (Goel & Mishra, 2018). The antibacterial activity of RS has been attributed to various mechanisms, including the degradation of DNA, impairment of cell division, oxidative damage to the cell membrane, and inhibition of enzymes involved in the electron transport chain (Ma et al., 2018). Gram‐negative bacteria, which have an external hydrophilic lipopolysaccharide (LPS) membrane, tend to be more resistant to hydrophobic compounds like TQ and RS compared to Gram‐positive bacteria. In Gram‐positive bacteria, the phospholipid bilayer membrane interacts with the hydrophobic components present in phenolic compounds, resulting in increased ion permeability, leakage of vital cell compounds, and disruption of the bacterial enzyme system, ultimately leading to cell death (Sandri et al., 2007). Additional factors that can contribute to the decreased antibacterial effectiveness of phenolic compounds in Gram‐negative bacteria include the presence of degradative and detoxifying enzymes in the periplasmic space and the existence of multidrug resistance pumps on their surface (Ma et al., 2018). On the other hand, Table 2 also demonstrates the antagonistic antibacterial effects between TQ and RS in all combination samples (ZN/PVA‐TQ2‐RS1, ZN/PVA‐TQ2‐RS2, ZN/PVA‐TQ4‐RS1, and ZN/PVA‐TQ4‐RS2). It has been proven that RS exhibits its intrinsic antioxidant properties through scavenging ROS. ROS is an intermediary substance generated during specific antimicrobial treatments, and its presence induces microorganism death (Tosato et al., 2018). Considering that TQ exerts its antimicrobial effects through the production of ROS, we can conclude that this phenomenon is linked to the antioxidant activity of RS on the ROS produced by TQ. However, some mechanisms have been proposed for synergistic antimicrobial effects: inhibition of protective enzymes, sequential inhibition of a common biochemical pathway, use of cell wall active agents to enhance the uptake of other antimicrobials, and combinations of cell wall‐active agents (Santiesteban‐López et al., 2007). The possibility of antagonistic effects increases with disruption of one or more of the mentioned mechanisms.

#### 3.4.2 Antioxidant properties

The DPPH assay is considered as a simple, rapid, and independent sample polarity method to evaluate the antioxidant capacity of bioactive compounds through radical scavenging screening (Salarbashi et al., 2014). Table 3 shows the DPPH scavenging activity of ZN/PVA nanofibers containing different concentrations of TQ with or without electrosprayed nanoparticles containing different concentrations of RS. The ZN/PVA nanofiber created without adding TQ and RS nanoparticles (CON group) had a slight radical scavenging activity (10.36%), which was probably related to the reaction of the residual free amino groups in ZN to form stable macromolecule radicals. Amino groups can form ammonium groups by absorbing a hydrogen ion from the solution (Heydari‐Majd et al., 2019; Siripatrawan & Harte, 2010). Some studies have attributed the antioxidant activity of ZN polymers to the presence of amino acids with phenyl groups such as proline in their structure (Leena et al., 2020). In addition, the high surface area of ZN/PVA nanofiber can provide more contact with the free radical solution and thereby inhibit them (Najafi et al., 2022). The DPPH scavenging activities of all treatments were significantly increased by increasing the concentration of TQ in nanofibers or RS in nanoparticles (*p* ≤ .05). Also, combined treatments showed more antioxidant activity than single treatments (*p* ≤ .05), with synergistic effects between TQ and RS (combination index>1). The highest antioxidant activity belonged to the ZN/PVA‐TQ4‐RS2 nanofiber, with 99.48% radical scavenging activity (*p* ≤ .05). In accordance with the present results, Jayan et al. (2019) showed an increasing trend in DPPH inhibitory activity of electrosprayed ZN nanoparticles with increasing RS concentration. They claimed that the encapsulation of RS inside the ZN matrix protected its antioxidant potential. Moreover, Leena et al. (2020) indicated similar results about the DPPH inhibitory activities of ZN nanofibers containing different concentrations of RS. In addition, other studies have also reported the similar concentration‐dependent manner of DPPH scavenging activities of different food packaging materials containing TQ or RS (Aminzare et al., 2022; Yousefizadeh, Hassanzadazar, & Aminzare, 2022). It has been proven that the antioxidant activity of RS is due to its two phenolic groups, which can stabilize free radicals formed on phenolic carbons with their resonance activity (Busolo & Lagaron, 2015). Moreover, RS can prevent further oxidative processes by inhibiting the generation of hydroxyl radicals (Truong et al., 2018). Kotora et al. (2016) reported that the antioxidant capacity of RS is due to the number and position of its hydroxyl groups. On the other hand, it has been suggested that the antioxidant activity of TQ is mainly due to its ability to scavenge carbon‐centered free radicals and reactive oxygen species and not due to chelating iron ions (Badary et al., 2003). TQ scavenges DPPH radicals through its redox characteristic and through a hydrogen donation mechanism (Yildiz et al., 2021). The synergistic antioxidant interactions between TQ or RS and other bioactive compounds have also been reported in previous studies (Aminzare et al., 2022; Skroza et al., 2015; Yousefizadeh, Hassanzadazar, & Aminzare, 2022). Several hypotheses have been proposed to explain the synergistic mechanism between antioxidants, such as new phenolic compounds or dimers formed between antioxidants with higher antioxidant activity; new intermolecular structure creation; diversity in phase distribution and solubility of antioxidants; regeneration of antioxidants with lower reducing power by antioxidants with higher reduction potency; and interactions between active ingredients that are not anticipated (Olszowy‐Tomczyk, 2020).

**TABLE 3:** DPPH scavenging activity of ZN/PVA nanofibers incorporated with TQ and/or electrosprayed RS nanoparticles (Mean ± SE).

Sample	Scavenging activity (%)	Combination index
ZN/PVA	10.36 ± 0.08^a^	–
ZN/PVA‐TQ2	20.53 ± 0.10^b^	–
ZN/PVA‐TQ4	39.58 ± 0.05^c^	–
ZN/PVA‐RS1	72.55 ± 0.11^d^	–
ZN/PVA‐RS2	89.22 ± 0.06^e^	–
ZN/PVA‐TQ2‐RS1	77.39 ± 0.11^f^	2.42 (S)
ZN/PVA‐TQ2‐RS2	93.66 ± 0.08^g^	2.81 (S)
ZN/PVA‐TQ4‐RS1	82.28 ± 0.07^h^	1.61 (S)
ZN/PVA‐TQ4‐RS2	99.48 ± 0.09^i^	1.81 (S)

*Note*: Values followed by different letters within the same columns are significantly different according to the Tukey's test (*p* ≤ .05).

Abbreviations: A, Antagonistic effect; Ad, Additive effect; S, Synergistic effect.

## 4 CONCLUSIONS

In the present study, ZN/PVA nanofibers containing 2 and 4% TQ were prepared using the electrospinning method and then electrosprayed with ZN nanoparticles containing 1% and 2% RS. The characteristics of the nanofibers were determined using SEM, FT‐IR, and TGA methods, and their in vitro antibacterial and antioxidant potentials were evaluated. Antimicrobial and antioxidant properties of nanofibers increased with increasing concentrations of TQ and RS. *S. aureus* was more sensitive than *E. coli* O157:H7 against all treatments. The combination of TQ and RES electrosprayed nanoparticles in ZN/PVA nanofibers demonstrated antagonistic antibacterial and synergistic antioxidant effects. The best results were obtained with the ZN/PVA nanofiber containing 4% TQ and electrosprayed with ZN nanoparticles containing 2% RS. According to the findings of the present study, biodegradable ZN/PVA nanofibers containing TQ and electrosprayed with RS nanoparticles are proposed as a novel active packaging material for the food industry. However, it is recommended to evaluate the cytotoxicity or hemolysis ratio of these food packaging materials in future studies.

## AUTHOR CONTRIBUTIONS

**Majid Aminzare:** Conceptualization (lead); data curation (supporting); formal analysis (lead); funding acquisition (lead); investigation (equal); methodology (lead); project administration (lead); resources (lead); software (equal); supervision (lead); validation (lead); visualization (lead); writing – original draft (equal); writing – review and editing (lead). **Saeideh Soltan Ahmadi:** Conceptualization (equal); data curation (lead); investigation (lead); methodology (supporting); software (equal); validation (equal); visualization (equal); writing – original draft (equal); writing – review and editing (equal). **Hassan Hassanzad Azar:** Funding acquisition (supporting); investigation (supporting); methodology (supporting); project administration (supporting); resources (supporting); supervision (supporting); validation (supporting); writing – original draft (supporting); writing – review and editing (equal). **Nasser Nikfarjam:** Conceptualization (supporting); data curation (equal); investigation (equal); methodology (supporting); validation (supporting); writing – original draft (supporting); writing – review and editing (equal). **Shahin Roohinejad:** Conceptualization (supporting); investigation (supporting); methodology (supporting); validation (supporting); writing – original draft (supporting); writing – review and editing (equal). **Ralf Greiner:** Conceptualization (supporting); investigation (supporting); methodology (supporting); validation (supporting); writing – original draft (supporting); writing – review and editing (equal). **Reza Tahergorabi:** Conceptualization (supporting); investigation (supporting); methodology (supporting); validation (supporting); writing – original draft (supporting); writing – review and editing (equal).

## CONFLICT OF INTEREST STATEMENT

The authors declare no conflicts of interest for this study.

## Data Availability

The data that support the findings of this study are available on request from the corresponding author. The data are not publicly available due to privacy or ethical restrictions.
